# Collaborative Autoethnography of Cancer Patients’ Dynamic Sense of Agency

**DOI:** 10.1177/10497323241285959

**Published:** 2024-10-18

**Authors:** Eeva Aromaa, Päivi Eriksson, Satu Koskinen

**Affiliations:** 1 Business School, 163043 University of Eastern Finland, Kuopio, Finland; 2 Free Researcher, Entrepreneur at Boardcoach, Joensuu, Finland

**Keywords:** cancer, illness management, sense of agency, collaborative autoethnography, patient story, COVID-19 pandemic, threat of war, Finland

## Abstract

Through collaborative autoethnography, we studied shifts in cancer patients’ sense of agency and the meaning of cancer during the diagnostic and treatment phases. This article contributes to the illness management literature by adopting sense of agency perspective that provides new understanding of retrospective interpretation of cancer patients’ agency. The authors’ experiences of receiving cancer diagnoses and a related, collectively written story illustrate how relational and contextual elements facilitate rapid shifts in cancer patients’ sense of agency and illness management. The findings illustrate shifts in the sense of agency as a collaborative and reflexive process between cognitive, emotional, and bodily constraints and adjustments. We demonstrate how shifts in patients’ sense of agency and respective changes in meanings attached to cancer were shaped by near ones, healthcare actors, and other cancer patients, as well as the COVID-19 pandemic and the fear of military conflict due to Finland neighbor Russia’s war on Ukraine. Furthermore, the study illustrates how shifts in sense of agency shape and are shaped by changes in the understanding of cancer as either a secondary issue, ambiguous stranger, travel companion, or enemy.

## Introduction

In the illness management literature, an increasing number of autoethnographies have contributed to understanding how cancer patients navigate the complexities they encounter after diagnosis. Previous autoethnographic studies have shed light on significant issues—such as objectification of patients in surgical contexts (Gross, 2012), existential challenges experienced during adjuvant chemotherapy (Greenhalgh, 2017), restoration of ruptured identities (Riessman, 2015), and fear of cancer recurrence—that impact everyday health competence (Horlick-Jones, 2011).

Despite normalization of patients undergoing various procedures to diagnose and remove tumors and metastases (Wan et al., 2013), our understanding of how patients construct their identities as agentic actors in response to challenges during diagnostic and treatment phases of care processes remains limited. Throughout the care process, patients encounter several complexities, including, but not limited to, stigmatized perceptions, pain, intense emotions, information gaps, unfamiliar medical jargon, and unforeseen crises in their daily lives (Brennan & Moynihan, 2004). Previous research has depicted illness management as a dynamic process influenced by shifts in diagnoses and treatments (Nowakowski & Sumerau, 2019), and social and material resources (Henshall et al., 2018). However, patients’ retrospective interpretations of their own agency and meanings given to cancer during various care stages remain largely underexamined. Therefore, we ask: How do relational and contextual elements facilitate shifts in cancer patients’ sense of agency and changes in their understandings of cancer?

This article contributes to the illness management literature on cancer patients (Greenhalgh, 2017; Gross, 2012; Horlick-Jones, 2011; Riessman, 2015) by adopting a “sense of agency” perspective. Understanding human agency necessitates distinguishing real-time experiences of agency from socially constructed meanings attached retrospectively to these experiences. The latter, conceptualized as sense of agency, refers to individuals’ retrospective perceptions of themselves as actors who can control and manage their own understanding, actions, and events (Acke & Meganck, 2022; Deans, 2019). By adopting the collaborative autoethnography (CAE) method (Chang, 2016; Chang et al., 2016), this article highlights how individuals’ sense of agency is constructed retrospectively, with the co-authors conducting CAE in relation to near ones, healthcare actors, and other cancer patients. Thus, CAE entails conducting autoethnography with others, enabling readers to access deeply personal health-related experiences that are elaborated and enriched collectively by multiple authors (Chang et al., 2016).

For patients grappling with serious illnesses, sense of agency holds paramount importance, as it profoundly shapes their ability to perceive themselves as competent in managing their illness, thereby impacting their future health and performance (De Luca Picione et al., 2017). In industrialized societies, cancer presents distinct challenges compared with other serious illnesses due to significant recent advances in diagnoses and medical treatments. However, the stigma and cultural perception of cancer as a fatal illness have remained, potentially diminishing the sense of agency among those diagnosed by positioning them as deviating from the normal (Shim et al., 2021).

Previous empirical studies on cancer patients’ sense of agency are scarce and tend to portray agency as a static phenomenon or dependent variable shaped by physical (Midtgaard et al., 2007) and psychological (Talebi et al., 2023) rehabilitation’s efficacy, as well as patients’ identity construction (Rees, 2017). The present study builds on prior illness management research by directing attention to shifts in patients’ sense of agency that both shape and are shaped by meanings attached to cancer.

Autoethnographic data often includes illness narratives (e.g., Brooks, 2011; Lahman, 2009). This paper builds on this research stream but highlights illness stories as collaborative constructions. Employing CAE as the methodological approach, the data comprise a collaboratively produced, analyzed, and written story of the first author’s bowel cancer journey. The analysis draws attention to contextual macro- and meso-level dimensions and social interactions that shape the retrospective construction of patients’ agency (Prasad, 2014).

The patient story encompasses experiences between September 2021 and April 2022, during which Finnish society experienced the COVID-19 pandemic and an increased threat of military conflict with neighboring Russia after launching its war against Ukraine. Leveraging insights from the sense of agency literature, we aimed to portray illness management among cancer patients as a contextual and relational process (Acke & Meganck, 2022; Deans, 2019) characterized by patients’ agentic positions that are connected to changing meanings that patients attach to cancer.

This study did not aim to generalize its findings to the broader population of those with cancer or other severe conditions but rather to understand nuances in cancer patients’ shifting agentic experiences and meanings that may manifest over time. However, each patient may interpret symptom fluctuations, diagnostic elements, and various treatments differently, as these are shaped by interpersonal relationships and societal contexts (Isaksen et al., 2003).

Rather than portraying cancer patients as heroic figures who can manage their illness singlehandedly (Gray & Doan, 1990), our study depicts patients as reflexive actors navigating a delicate balance between gaining and losing their sense of agency.

## Cancer and Sense of Agency as Socially Constructed Phenomena

Rather than conceptualizing cancer solely as a biological condition (Willig, 2011) and agency as a fixed, realist phenomenon (Frie & Reis, 2005), we adopted a social constructionist perspective. In viewing cancer and sense of agency as socially constructed phenomena, cancer patients can be understood as reflexive actors who continually reassess their perceptions of cancer and their own agency in response to new definitions and situations enacted by various actors (Weick et al., 2005). Shifts in understanding cancer and agency are linked intricately to changes in medical knowledge, interactions among diverse social actors, social norms, and societal circumstances (Nowakowski & Sumerau, 2019).

In our study, various meanings that a patient attaches to cancer and the patient’s perceptions of agency are shaped by how medical authorities, healthcare professionals, fellow patients, and the media individually and collectively define health, illness, and the patient’s role within established societal frameworks (Barker, 2010; Sharf & Vanderford, 2003). In this study, cancer patients are viewed as social actors who are familiar with the cultural and societal contexts in which they reside and can navigate various situations. As reflexive actors, their actions oscillate between spontaneity and strategy, depending on the context (Ng & Kidder, 2010).

This article adopts sense of agency perspective (Acke & Meganck, 2022; Deans, 2019) to comprehend illness management as a dynamic phenomenon. Sense of agency is characterized as individuals’ experiences of themselves as agentic actors who can control their actions and influence events in the external world (Haggard, 2017; Haggard & Chambon, 2012). Although agency often connotes autonomy and independence (Adler et al., 2008), Acke and Meganck (2022) described agency as a relational and contextual phenomenon, highlighting how patients interpret their actions in relation to others, such as healthcare practitioners, to organize their experiences and attribute meanings to their actions (Seilonen & Wahlström, 2016). A relational perspective challenges the binary approach to agency, recognizing individuals as neither purely active agents nor passive objects subject to power structures (Bamberg, 2011). Gallagher (2012) suggested further that contextual elements shape how actors perceive their capacity to take action in a given situation.

Rooted in agential phenomenology, feminist studies, and anthropology, agency is perceived as a sense of empowerment (Campbell & Maynell, 2010). Accordingly, sense of agency fluctuates based on our ability to consider alternative courses of action, pursue intended goals (Palmer, 2007), take responsibility for our actions’ outcomes (Acke & Meganck, 2022), and perceive our actions as meaningful, with intended impacts on the world (Deans, 2019).

## Collaborative Autoethnography

CAE enables readers to access deeply personal health-related experiences, which are collectively elaborated and enriched by multiple authors (Chang et al., 2016). Discussing and comparing experiences help autoethnographers analyze contextual and relational elements of human understanding and action, instead of focusing only on individual experiences, such as inner sensations and feelings (Chang, 2016; Chang et al., 2016). Individually conducted autoethnographies sometimes suffer from a lack of critical distance when the researcher and subject are the same person. However, CAE benefits from the diversity of experiential and scholarly perspectives among researchers, enhancing analytical and interpretive components (Lapadat, 2017; Richards, 2008). Therefore, CAE reduces the likelihood of criticism over narcissism, self-indulgence, or lack of rigor (Lapadat, 2017).

CAE emphasizes a reflexive stance toward collective knowledge production, characterized by researchers moving their positions along a continuum between insider and outsider roles during the various stages of the research process (Ellis & Rawicki, 2013; Eriksson et al., 2012). These shifts in the researcher’s position happen in relation to data production, analysis, and writing. Some researchers may play a legitimate role in a CAE by making their voices heard in another narrator’s story (Díaz-Kommonen, 2017). In this case, their voice remains more silent to the reader, even if recognizable to co-ethnographers. Being reflexive, negotiating, and reporting on researchers’ roles during the research process enhance qualitative research trustworthiness and rigor (Eriksson & Kovalainen, 2015).

CAE as a research method raises questions about relationships and mutual trust between authors who, in the health and illness context, share their intimate experiences, vulnerable emotions, and identity struggles during the collaborative research process (Chang et al., 2016). Thus, autoethnographic research calls for somewhat close relationships between authors while maintaining a critical and reflexive stance toward experiential knowledge. In this article, the first author, Eeva, and third author, Satu, share a common background, earning PhDs in business studies at Finnish universities. Eeva has a PhD in economics and business administration and is a postdoctoral researcher at a Finnish university’s business school. With a background in physiotherapy and a master’s degree in health sciences, she previously worked as a researcher at a national health institution. Satu has a PhD in management and leadership and serves on several companies’ boards. During the past decade, Eeva and Satu became acquainted during discussions at conferences, including a weeklong conference trip together. In September 2021, both were diagnosed with bowel cancer, had surgery, and received chemotherapy in different Finnish hospitals. Currently, both are back at work and enjoying life like before. In the wake of their diagnoses, Eeva and Satu had meaningful conversations in October 2021 while trying to deal with their plight, providing this article’s thrust.

The second author, Päivi, is a professor of management and an expert in multiple qualitative methodologies (Eriksson & Kovalainen, 2015), including autoethnography (Eriksson, 2013), reflexivity in ethnographic research (Eriksson et al., 2012), and insider (emic) research perspectives (Laukkanen & Eriksson, 2013). Päivi’s mother was diagnosed with stomach cancer in 2019 and died during the pandemic in 2020. Between 2018 and 2020, Päivi was the main caregiver for her mother, with lived experiences on the struggle to maintain a sense of agency, both for her mother and herself—one of this paper’s key empirical and theoretical aspects. In CAE, including co-authors with different kinds of insider perspectives (Laukkanen & Eriksson, 2013; Spiers, 2000) allows for integrating shared experiences with contextual elements, thereby offering unique insights and generating new knowledge on social life (Chang et al., 2016).

In addition to her qualitative research expertise and experiences caring for a cancer patient, Päivi’s role on the author team was as a mentor, which she has performed with about 20 postdoctoral researchers over the past 25 years, among other mentoring tasks at the university. Päivi was the main supervisor for Eeva’s doctoral thesis and thereafter continued as her postdoc supervisor. Both worked in the same research group at the business school going back 20 years, with Päivi as leader and Eeva as a group member. They worked together on about 10 research projects, attending dozens of conferences and other events abroad, including five in India and four in Canada, while sharing experiences on professional and private issues. Päivi and Satu have known each other for about 10 years due to Päivi’s interest in Satu’s dissertation on leadership in the dyadic CEO–chair relationship, during which Satu asked for Päivi’s advice on academic issues. They also travelled to conferences together.

### The Collaborative Research Process, Including Data Generation and Analysis

The two-stage CAE was based on sharing and exploring personal experiences related to illness and then transforming them into scholarly output that adds to wider academic and societal understandings. While this article focused on Eeva’s story, the iterative and reflexive analysis and feedback provided by Päivi and Satu functioned as crucial “mirrors” for purposes of refining personal experiences and conceptualizing them into an academic contribution. Next, we explain our two-phase CAE process.

#### Phase 1: Crafting, Sharing, and Analyzing Personal Illness Stories

##### Individual Story Development

From October 2021 to April 2022, Eeva and Satu discussed their personal experiences as bowel cancer patients while meeting every 3 weeks on Teams online. This regular engagement allowed for deep examination of personal illness experiences (Ellis & Rawicki, 2013), including, but not limited to, cancer treatments, changing emotions, and identity struggles. In Finland, patients can choose where they want to receive healthcare from among the nation’s facilities, covering primary and specialized care. Satu and Eeva were treated at different hospitals with different clinical practices, which enriched their discussions. Their emails and WhatsApp messages with each other, individual notes, diaries, and records from MyKanta patient information system served as supplementary data with their conversations. Through MyKanta, citizens have access to records on their healthcare appointments, prescriptions, and laboratory tests. During individual story development, Satu relied on her notes, whereas Eeva utilized emotional recall (Bochner & Ellis, 2016), to enhance recollection of their stories’ details. Writing individual stories enabled iterative reflection, which was crucial for capturing their experiences’ nuanced complexity.

##### Sharing and Enriching Stories

Eeva and Satu shared their written stories with Päivi, whose early involvement was more as an outsider mentor, but she soon became an insider (Ellis & Rawicki, 2013) because of her experiences as a caregiver for her mother. The intense conversations with Päivi and her scholarly feedback added a fresh reflexive layer to the analysis, enriching the nuances in Eeva’s and Satu’s stories, and highlighting their differences. Päivi sensitized Eeva and Satu to differences in how they constructed agency and managed their illness. Eeva’s story focus was on shifts in her agency that were shaped by contextual elements of the pandemic and the changing security–political situation in Finland, while Satu’s story illustrated a need for control over illness experiences, focusing on interactions with healthcare professionals.

##### Collaborative Analysis

As the three authors collaborated to analyze the key plots iteratively, themes and categories in Eeva’s and Satu’s stories facilitated a more nuanced and structured analytical understanding, helping to identify common themes and unique experiences, and adding initial conceptualizations to the stories. The key plot in Eeva’s story concerned her agentic ups and downs. The main themes focused on changing cognitive, emotional, and bodily experiences shaped by societal elements. The key plot in Satu’s story focused on maintaining control over illness experiences. The main themes comprised routines and practices enacted by healthcare professionals and herself.

##### Decision to Draft Two Articles

Differences in key plots and main themes in Eeva’s and Satu’s stories identified in the collaborative analysis led to the collective decision to draft two articles instead of one due to the richness of the experiences captured, as well as the unique theoretical potentials. Collins and Stockton (2018) emphasized that careful selection of theoretical perspectives is a fundamental task in enhancing epistemological rigor in qualitative research, and we realized that Eeva’s and Satu’s stories deserved separate attention.

#### Phase 2: Turning Personal Experience Into a Scholarly Contribution

##### Deepening Collaborative Analysis and Experimenting With Different Theoretical Frames

Eeva and Satu conducted the first version of their literature review, which Päivi elaborated further. During a 1-month research visit in Canada, Päivi and Eeva reflexively discussed several related literatures, which led to re-analysis of Eeva’s story through the theoretical lenses of sense of agency (Acke & Meganck, 2022; Deans, 2019) and illness management (Greenhalgh, 2017; Gross, 2012; Horlick-Jones, 2011; Riessman, 2015) to strengthen the structured analytical approach to it. The aim was to theorize personal experience while making it relevant and relatable through the chosen conceptual frameworks. The analysis was conducted through direct interpretation, relying on procedures such as intuition, mind mapping, and memo writing—a process that underscores researchers’ role as self-reflexive interpreters of the data (Eriksson, 2013; Eriksson et al., 2012). Contextual data from Finnish and international media outlets, publications from Finnish health and social care trade unions, and press releases (see The Finnish Government, 2020) informed the analysis.

##### Continuous Critical and Informed Feedback

Having Päivi as a critical reader added an important reflexive layer to the research process, ensuring clarity, depth, and rigor in the analysis and theoretical work. This role involved questioning pre-assumptions and biases, and providing feedback, which was crucial in refining the manuscript for publication. During reflexive discussions with Päivi, Eeva noticed that the manuscript constructed a dichotomic understanding of agency as an increasing and decreasing phenomenon. Furthermore, Päivi highlighted a new key finding—the changing meaning of cancer—which was implicit in the story, but could be enriched. Thus, Päivi encouraged Eeva and Satu to discuss the changing meaning of cancer in the analysis of Eeva’s story.

##### Collaborative Revisions

The authors’ iterative fine-tuning of the findings ensured the manuscript’s quality and coherence. In terms of justification of data inclusion and exclusion, we included data based on its relevance to the research questions. We discussed and justified to each other the reasons for including or excluding specific pieces of data. After several phases of collaborative work, the manuscript included an analysis of shifts in the sense of agency and the meaning of cancer, highlighting how these facilitated cognitive, emotional, and bodily adjustments in illness management—all contributing to the literatures on sense of agency and illness management. The collaborative effort described here reflects the postmodernist and postfeminist way of conducting research, aiming to portray the illness experience as ambiguous, fluid, multi-vocal, and collectively shaped (Ehlers & Krupar, 2012).

### Ethical Considerations

Autoethnographies represent and appropriate the voice of self and others. Researchers’ duty is to protect the rights of all these voices (Chang, 2016). Autoethnographers must follow procedural research ethics (Lapadat, 2017) that include acquiring institutional approval and participants’ informed consent while ensuring their confidentiality. In autoethnography, conducting anticipatory ethics (Tolich, 2014) by acquiring informed consent from participants before collecting data is problematic. Tolich (2010) has questioned the practice of seeking informed consent after writing an article as problematic and potentially coercive, placing research subjects in an unfair power position by making them feel compelled to volunteer. Our research followed Finnish National Board on Research Integrity guidelines (Kohonen et al., 2019). This article is part of the project “Cancer Experiences During Unsettled Times: Collaborative Autoethnography,” which was approved by the University of Eastern Finland Committee on Research Ethics (Statement 33/2022, dated September 26, 2022). In our self-assessment report, we carefully considered the ethical challenges from our research, organized around issues of how to protect our privacy and that of those mentioned in the publication due to their actions, opinions, or understandings. Drawing on relational ethics (Denshire, 2014; Ellis, 2007), the procedures we followed are explained below.

#### Procedures to Protect Those Mentioned in the Story

Ellis (2007) asked autoethnographers to consider their responsibilities to intimate others who are characters in the stories we tell about our lives. The procedures we took to protect those mentioned in the story included omission of unique identifiers and place names, and the use of pseudonyms (Lapadat, 2017). We protected the hospital’s medical staff by not disclosing the name of the hospital. We anonymized the names of fellow hospital patients and the cancer patient in the home region and omitted identifiers (someone telling a joke in the home region). Even if the quality of the relationship between them and Eeva was omitted to protect individuals, the story presents rather authentic accounts by these individuals, illustrating how they caused mental strain for Eeva. She discussed the story’s content with these individuals, and these conversations turned into a discussion on how the rapidly changing security–political situation in Finland caused distress, made real in their discussions. These individuals did not see any harm in publishing the story. With the family members—the father and the spouse—the connection between them and Eeva is easy to identify, and they could recognize themselves from a written account (Ellis, 2007). Eeva discussed the story to them, and both agreed that the content described their “lived life as it was” and that they could not see any risk to them.

#### Procedures to Protect the Authors

As autoethnographers, our work can affect ourselves in unforeseen ways. Reading and writing personal and traumatic stories can elicit strong emotions and stir up unresolved grief or repressed memories (Denshire, 2014). Simultaneously, the collaborative process can be therapeutic, as it helps authors (and readers) make sense of their lives, and it can reduce the sense of isolation regarding traumatic experiences. Eeva, Päivi, and Satu discussed this collaborative method of sharing, elaborating, and interpreting illness experiences that fostered empathetic listening between them and a deeper understanding of themselves and the cultivation of positivity in a challenging life situation (Ellis, 1999; Watfern et al., 2023). This process served therapeutic purposes (Richards, 2008). Even though the process supports inclusion inside the research team, when researchers publish stories without anonymity protection, they risk stigmatization, negative judgments by colleagues, and undesired career consequences (Lapadat, 2017). These potential consequences were considered but not viewed as potentially harmful for the authors. Bowel cancer is a common disease in Western countries (Sharma et al., 2022) and is discussed regularly in Finnish media. Bowel cancer screenings started in Finland in 2022—a year after Eeva received her delayed diagnoses. Furthermore, to respect each other’s dignity and autonomy, we were free to cease participation in the study at any time. In this case, other authors will not use unpublished collaboratively produced illness stories.

#### Procedures to Protect the First Author and Her Family

Autoethnographers often use their own names in publications, making it difficult to protect the anonymity of others mentioned in the story, such as family members (Ellis, 2007; Tolich, 2014). The first author, whose illness story is presented in this article, discussed the content with her adolescent children and spouse. The oldest child, who studies at the university, read the manuscript.

## Findings: Dynamics of Cancer Patients’ Sense of Agency and Changing Meaning of Cancer

Before outlining our analysis of cancer patients’ sense of agency, we discuss Eeva’s early experiences, including delays in cancer diagnosis exacerbated by the COVID-19 pandemic and other contextual factors. We offer a contextual background on how and why the patient experienced an increased threat of war in a particular manner. These experiences serve as a backdrop to understanding and contextualizing glimpses of the illness management process from the perspective of a shifting sense of agency in Eeva’s case.

Eeva’s story provides an illustrative case of a patient who, as a physiotherapist with a degree in health sciences, had some professional expertise in health and illness prior to her own diagnosis. The discussions with the co-authors led to understanding that Eeva’s story is shaped deeply by two unexpected crises in Finnish society—the COVID-19 pandemic and Russia’s war against Ukraine—which impacted her experiences as a person living with a cancer diagnosis, particularly as the prognosis worsened during this time.

Reflecting on Eeva’s life during the past 5 years, Päivi and Satu brought up how getting a cancer diagnosis was a prolonged process, as potential cancer was a secondary issue in Eeva’s life. As early as 2018, she sensed something was amiss with her health. Despite receiving a referral to undergo an endoscopy from an occupational health physician in spring 2018, she deferred the hospital visit due to the complexity of caring for family members with health issues. There was no room for additional negative news. By February 2020, when the pandemic reached Finland, Eeva was in the final stages of completing her doctoral thesis. She intended to schedule a doctor’s appointment after defending her thesis in September 2020 but failed to do so. Reports of the strain on the Finnish healthcare system due to the pandemic led her to downplay her own health concerns.

Summer 2021 was a turning point in getting a diagnosis, as Eeva understood that the pandemic would not end quickly. Contemplating her role as caregiver for her family, including her elderly father, she realized she could no longer delay hospital exams. Acting on a new referral, she scheduled an appointment at the endoscopy unit. However, she cancelled it due to flu-like symptoms. As COVID-19 testing was challenging to access, and nurses in hospitals were in short supply, she refrained from taking any risks that potentially could expose medical staff to the virus.

Finnish media reports highlighted how the pandemic had led to delays in cancer diagnosis and treatment, which allowed tumors to progress (Page et al., 2022). Eeva’s case exemplifies how media discourse influenced her decision to postpone diagnostic procedures. The analytical process between authors illustrated how Eeva initially underestimated her health condition’s urgency. Although she later challenged the healthcare overload discourse previously adopted uncritically, we recognized retrospectively that she acted like an excessively cautious person who feared transmitting the virus to medical staff.

After the diagnosis, Eeva’s health and illness situation was affected further by another unexpected threat when Russian troops attacked Ukraine at the end of February 2022, causing great alarm in Finland, which shares a 1300-kilometer (810-mile) border and some common war history with Russia.^1^ Whereas the previous part of Eeva’s background story was elaborated and enriched collectively during the research process, this part of the story takes one step from collective reflexivity toward individualistic reflexivity (Eriksson et al., 2012). This shift in the epistemic stance might reflect some sort of collective worry and silent fear that the authors experienced. At the time of collaborative production of Eeva’s story, Finland’s largest digital media outlet, Ilta-Sanomat, reported that a new age of danger had begun in Finland. All three authors agreed that this new threat of war, magnified in the media, affected Eeva’s experiences due to her background of living near a large military base during her early years and because some of her family members and friends still live in that area.

Eeva told Päivi and Satu retrospectively how, during the weeks following the Russian attack on Ukraine, she often visited her childhood home region near the military base. As a young adult, she had worked inside the base and played sports there for several years. During her visits, she became conscious of how strongly her identities, deep emotions, feelings of safety, and continuity of life were connected to that base and the people living there, even if she was not fully able to verbalize all nuances during the data elaboration process. In her home region, she was surrounded by old war stories and rumors about new plans in the area. A fear of a possible attack often was expressed through jokes, like this one: “I told my relatives living in the western part of Finland that in case of an attack, they don’t need to call me. Living in a place like this …. I’d be a pancake in a minute!” Even though she found herself laughing with others, a silent fear washed over her. In her mind, the threat of war seemed closer than ever before in her life, and she did not discuss this openly with Päivi and Satu.

Simultaneously, Eeva found herself struggling with her identity due to the diagnosis. These struggles were shared with Satu and elaborated in joint discussions. Challenging for Eeva was that elderly people positioned her as a patient. From their perspective, Eeva’s life would be worse in the future with limited opportunities. Taking the highest possible doses of cytotoxins at that time made Eeva feel even worse. She thought about previous wars’ consequences on her life. Both of her grandfathers served in past wars, and one lived with her family when she was a child. Living near the military base kept her grandfathers’ traumas alive. Her father lacked basic education in his early years because the school in the rural region was closed for several years due to evacuated people living in the school building. Even though Eeva’s father later gained access to study at the university, Eeva said he lacked self-confidence to do so.

The following four episodes of the story illustrate how relational and contextual elements shaped shifts in Eeva’s sense of agency and the meaning of cancer during the diagnostic and postoperative phases. The episodes and their interpretations demonstrate the cancer patient’s agency as a reflexive and dynamic process between cognitive, emotional, and bodily constraints and adjustments.

### The Preliminary Diagnosis

#### Cancer as an Ambiguous Stranger

The first episode that follows is illustrative of the shift that happened in Eeva’s agentic position from an object of medical examinations to an inquisitive and knowledgeable patient who contributes to her own care. The episode demonstrates how the shift in agency shapes and is shaped by understanding cancer as an ambiguous stranger. Together, Eeva and Satu talked about the importance of actively seeking medical information and articulating both medical and experiential knowledge about diagnostic procedures and bodily sensations to their family members and healthcare professionals that facilitated emotional and cognitive adjustment, depicted in the episode below.

**Episode 1.** In September-October 2021, the clinical examinations at the hospital progressed swiftly, adhering to the recommended standards of care for cancer patients in Finland. During the initial two weeks following the first endoscopy, I found myself visiting the hospital almost daily, which instilled in me a sense of being cared for by the healthcare system. However, in the initial days, despite receiving detailed explanations of the procedures, I felt like an outsider, as my body underwent medical examinations at such a rapid pace that my mind struggled to fully comprehend the content and results. Although another patient with a similar preliminary diagnosis had been advised by the doctor not to access their MyKanta patient information system records, I felt compelled to understand the procedures and results to avoid feeling detached from my own health and illness. Through online research, I familiarized myself with cancer terminology, enabling me to discern between positive and troubling news. I learned to interpret the multitude of blood test results. My ability to explain my situation and the hospital procedures to my family brought them a sense of relief.

On one occasion, upon arriving at the hospital, I encountered technical issues with the medical records. Despite this setback, I remained proactive in advocating for myself. At the endoscopy unit, I informed the gastroenterologist about metallic clips in my gut that may have contributed to the previous day’s failed magnetic resonance imaging. Following the endoscopy, I experienced intense neck-radiating nerve pain, making it difficult to remain stationary. At the gynecological unit, I engaged in negotiations with a nurse regarding the possibility of delaying my scheduled examination. Describing the pain sensations in detail and quantifying my discomfort, I successfully convinced the nurse of the severity of my condition. Surprisingly, I was granted permission to delay the examination, providing immense relief. By expressing my needs and concerns, I felt heard and valued as a patient.

Experiencing the preliminary diagnosis and awaiting further information regarding future diagnostic investigations often entail prolonged periods of uncertainty and anxiety (Samuelsson et al., 2018). However, the episode above demonstrates how the diagnostic process unfolded swiftly, resulting in a sense of being cared for, but also creating a feeling of detachment from her own care. Together, we co-authors noticed how the examinations’ rapid pace left Eeva feeling like a passive recipient, unable to fully comprehend the ambiguous medical language and diagnostic procedures involved. In response to feeling disconnected from her own care, Satu pointed out how Eeva sought out medical information to empower herself and better manage her emotions and those of her family members (Hochschild, 1983). Still understanding cancer as an ambiguous stranger stimulated Eeva’s proactive behavior, enabling her to participate actively in her own care and alter treatment routines in response to severe pain. Her experience aligns with Schulman‐Green and Jeon’s (2017) findings, highlighting how increased knowledge of care procedures enhances communication, self-efficacy, and the ability to navigate transitions, ultimately alleviating uncertainty and anxiety.

#### The Postoperative Phase

Transitioning to the postoperative phase, a shift in the patient’s sense of agency is evident as bodily sensations come to the forefront of their experience (Nowakowski & Sumerau, 2019). The second episode below illustrates a transition in the sense of agency from reliance on medical professionals for information to a resilient actor who can interpret bodily sensations independently. It demonstrates how the shift in agency shapes and is shaped by understanding cancer as an ambiguous stranger. Employing cognitive–emotional adjustments, such as utilizing imagination to normalize bodily sensations following the operation, underscores Eeva’s evolving sense of agency and ability to navigate the healing process:

**Episode 2.** In the days following the extensive six-hour operation, I experienced a shift in my sense of agency as I grappled with unfamiliar bodily sensations and sought to interpret them independently. Agreeing to reduce the epidural anesthesia dosage, I began to sense indistinct movements and pressure in the operated area, disrupting my sleep at night. Describing these sensations proved challenging.

During the doctor’s rounds, I said my organs have been salsa dancing all night long. I expected that a doctor would be able to read between the lines that I needed an explanation for my sensations. The doctor answered by looking at his papers: ‘The potassium levels are at the normal level’. It was a confusing situation. However, the doctor did not address my symptoms, so I thought they were normal postoperative sensations. Understanding these sensations required me to rely on my imagination. I developed sympathy with my healthy organs, how they might feel and think. They had lost some of their neighbors. I imagined my liver and gallbladder talking to each other. They felt sorrow after losing their late friends, but also felt honored having an opportunity to meet a surgical robot representing state-of-the-art health technology. They haggled over who will take care of the tasks of the lost organs. These imaginary scenarios helped me understand these weird sensations as being normal after going through a major operation.

The second episode demonstrated how Eeva navigated the days following the operation, confronting unfamiliar bodily sensations while still understanding cancer as an ambiguous stranger, but now prompting a profound shift in her sense of agency. Eeva and Satu discussed how verbalizing your sensations after surgery is not easy. For Eeva, this posed a challenge, prompting her to resort to metaphorical language during a doctor’s round, hoping to convey the urgency of her need for explanation. Unfortunately, the episode illustrates how the doctor’s response was disappointingly clinical, focusing solely on biochemical parameters common among medical specialists (Wong et al., 2020) without directly addressing Eeva’s symptoms. Street et al. (2009) emphasized that successful clinician–patient communication can empower patients to manage their health and illness.

The joint interpretation was that the setback Eeva felt made her seek solace in imaginative empathy, which is rarely described in previous literature (e.g., see Semino & Demjen, 2017), by envisioning a dialogue among her remaining healthy organs as they coped with the absence of their surgically removed counterparts. By reading previous literature, the authors agreed that this creative exercise might have helped Eeva normalize her experiences and articulate a range of emotions stemming from surgery to do grief work (Gabay, 2021) after losing several organs and parts of them during surgery. In the analysis of this episode, Eeva emphasized how using imagination let her celebrate benefitting from cutting-edge medical technology—a surgical robot—strengthening her optimism to be cured (Vatandoost & Litkouhi, 2019). Furthermore, Eeva commented how this internal dialogue served as a source of comfort, solace, and amusement during solitary nights, underscoring her growing capacity to interpret and make sense of bodily sensations autonomously.

#### As a Travel Companion

The third episode below illustrates a shift in sense of agency, transitioning from a decisive decision-maker to a reflexive actor who can find new meanings in her experience with cancer as a travel companion. Satu and Eeva shared how amid the pandemic, restrictions on public gatherings presented challenges for cancer patients seeking to maintain their physical and mental well-being. This episode below highlights how defying public opinion to avoid social gatherings allowed Eeva to reconceptualize health and illness as interconnected aspects of life, supporting her cognitive and emotional adjustment in the face of a worsened cancer prognosis. Päivi agreed that the interpretation about the change in the meaning of cancer is an intrinsic one in terms of managing health and illness.

**Episode 3.** Receiving the news of metastasis in December 2021 marked a turning point, leading me to commence combination treatment with oxaliplatin and capecitabine. One of the side effects of oxaliplatin, cold sensitivity, became particularly pronounced during the harsh winter temperatures, causing discomfort and constriction in my throat whenever I ventured outdoors. With outdoor exercise no longer feasible, I grappled with the decision on whether to exercise at home or brave the limited-capacity gyms open under COVID-19 restrictions.

Amidst the backdrop of a worsened prognosis and societal pressure to minimize public gatherings, I found myself torn between the desire for human connection and the responsibility to adhere to pandemic guidelines. However, an encounter at the gym with a woman who, like me, was going through cancer treatments reshaped my understanding of health and illness. Seeing her hairless body moving in relation to other bodies blurred the boundaries between health and illness, prompting me to view cancer as a companion rather than a foe to be feared.

This third episode above underscores the cognitive and emotional adjustments necessitated by changes in prognosis (Charmaz, 1983), particularly in the context of navigating uncertainty amid the pandemic. As cancer patients grappled with uncertainties surrounding medical care (Schneider et al., 2023) and leisure activities (Wilke et al., 2021) during this time, Eeva found herself confronting societal norms and choosing to act in a way that aligned with her evolving understanding of health and illness. Like Nowakowski (2016), her experience highlights how our bodies serve as a conduit for reframing and navigating our perceptions of health and illness, leading to profound cognitive and emotional shifts. In Eeva’s case, witnessing bodies moving at the gym facilitated a transformation in her perception of cancer, shifting from an ambiguous stranger to a companion on life’s journey.

#### As an Enemy

The fourth episode below illustrates a shift in sense of agency from enthusiastic peer support to a vulnerable and exposed person—a dramatic, temporary turn in illness management caused by the changed security–political situation in Finland. Furthermore, we co-authors agreed that the episode highlights a change in the meaning of cancer from a travel companion to an enemy that a patient must fight against.

**Episode 4.** In late February 2022, against the backdrop of escalating tensions following the Russian attack on Ukraine, I found myself grappling with a profound shift in my sense of agency and the meaning of cancer. Visiting my childhood home region, located near a large military base, I was asked to share my experiences with a fellow patient, Risto, who was soon to undergo surgery for bowel cancer. However, as we discussed the possibility of conflict escalation, the tranquillity of my home region was shattered by the specter of war. Despite reassurances from older family members, the threat of conflict loomed large, casting a pall of distress over me.

As I demonstrated postoperative movements to Risto in the familiar surroundings of my childhood home, the weight of history bore down on me. Learning that the region had once been a battleground during the previous war, I couldn’t shake the intense fear that gripped me. The pain from my wounds seemed to intensify, echoing the anguish of past generations who had endured wartime hardships. Despite my efforts to offer guidance to Risto, I couldn’t shake the vulnerability that now consumed me.

In the ensuing weeks, my sense of vulnerability only deepened as rumors of military plans swirled around me. My spouse’s commitment to swift military service in the event of conflict heightened my anxiety further, leaving me feeling like a bystander in my own life. The doctor recommended not to continue oxaliplatin treatment due to throat symptoms and to take capecitabine tablets only. With three children relying on me, the stakes felt unbearably high, amplifying my fears of an uncertain future. Yet, in the midst of uncertainty, I found solace in the resilience of the human spirit and the unwavering determination to face whatever challenges lie ahead.

The fourth episode above illustrates how contextual elements may facilitate relatively rapid shifts in the cancer patient’s sense of agency and illness management. Meeting with peer patient Risto in the context of armed attack made by a neighboring country with which our country shared some common military history underscored the profound impact of the rapidly changing security–political situation on Eeva’s sense of agency and how she perceived her illness. Our discussion against the backdrop of impending conflict forced Eeva to confront the fragility of life and the stark reality of her own mortality.

Regarding this episode, Päivi pointed out how sharing her postoperative journey with Risto as the weight of history hung heavy in the air (Nurmi, 1988) reminded her of the sacrifices made by those who came before us. In the episode, the echoes of wartime struggles reverberated through Eeva’s senses, amplifying her fear and uncertainty about the future. Despite her attempts to offer guidance, she couldn’t shake the feeling of helplessness that enveloped her, leaving her adrift in a sea of uncertainty. Furthermore, the episode illustrates how in the weeks that followed, the specter of war loomed large, casting a shadow over Eeva’s already fragile sense of security. Rumors of military plans and the prospect of conflict only served to deepen her anxiety, leaving her feeling powerless in the face of forces beyond her control. With Eeva’s spouse’s commitment to military service adding to her worries, she found herself grappling with the enormity of the challenges ahead.

Amid these tumultuous events, her decision to discontinue oxaliplatin treatment due to harmful side effects marked a pivotal moment in Eeva’s journey. As she sought to optimize her chances of defeating cancer, the disease ceased to be a mere companion and became a formidable adversary. Faced with the dual threats of illness and impending conflict, the episode above illuminates how her sense of agency wavered, leaving her feeling vulnerable and exposed, as the meaning of cancer shifted yet again, from a travel companion to an implacable foe.

## Discussion and Conclusions

This study highlights the dynamic nature of cancer patients’ sense of agency and their constructions of illness management. In a two-phase CAE process, we enriched and wrote one patient’s story to analyze how agentic action is constructed and influenced socially by various actors and societal contexts.

Prior research often conceptualizes agency as a static phenomenon studied as a dependent variable (Midtgaard et al., 2007; Rees, 2017; Talebi et al., 2023). In our study, we captured agency as a dynamic phenomenon that shapes and is shaped by changes in the meaning of cancer. Research on illness management understands meaning making in the context of patients’ existence and the purpose of life (Vachon, 2008). Often, meaning making is connected to religious and spiritual beliefs (O’Connor, 1990).

Our findings demonstrate how shifts in a patient’s sense of agency intertwined with meanings of cancer, morphing it over time from a secondary issue to an ambiguous stranger, then travel companion, and finally an enemy. This demonstrates how cancer can convey several meanings that shape and are shaped by shifts in cancer patients’ agency in relation to social, relational, and contextual elements. From the perspective of cancer care, all four meanings pose consequences for the patient, healthcare system, and society. For Eeva, understanding cancer as a secondary issue delayed diagnosis, required more radical surgery and a longer inpatient care period at the hospital, and could have led to a poor prognosis. However, understanding cancer as an ambiguous stranger stimulated learning behavior and led her to seek solace in imaginative empathy. Then understanding cancer as a travel companion supported her cognitive and emotional adjustment in the face of a worsened cancer prognosis. Finally, understanding cancer as an enemy left her feeling vulnerable and exposed.

In the health communication field, cancer metaphors and expressive metaphors comprise a rather established research area. In the Western world, cancer is a commonly used military metaphor used to describe an enemy, and the fight against cancer originated from a time when treatments were inadequate (Skott, 2002). Journey metaphors emphasize cancer’s impact on the patient’s life journey, leading to spiritual growth (Harrington, 2012). As a riddle or puzzle metaphor, it becomes a complex disease that needs to be treated, including scientific methods (Williams Camus, 2009). Our study demonstrates how different meanings attached to cancer, even if they resonate with well-known cancer discourse, are subjective and nuanced interpretations connected to various agency positions and are made real in a wide range of activities. The topic warrants further research.

In relation to previous literature, our study has elicited new findings on how the COVID-19 pandemic has impacted patients’ sense of agency. Recent research has demonstrated how existing health policies impacted health sector strategies that led to delays in diagnosis and treatment during COVID-19 (Riera et al., 2021). Adopting the patient perspective, our study demonstrates how media discourse on healthcare overload and a patient’s fear of exposing medical staff to the virus delayed her cancer diagnosis. However, prior research has reported how patients did not seek care due to fear of being infected with the virus (Sutcuoglu et al., 2023). Previous studies on the benefits of physical exercise have focused on patients’ physical and mental conditions, and clinical outcomes (Newton et al., 2020). Our study demonstrated a change in the meaning of cancer as a result of a patient participating in group exercise. Further studies on patients’ exercise and rehabilitation would benefit from adopting meaning-making perspectives to understand micro-mechanisms connected to patients’ subjective health experiences.

After hospital discharge, bowel cancer patients often feel vulnerable due to their surgery and fragile life situation. Thomsen and Hølge-Hazelton (2017) found this transition to everyday life heightened their vulnerability more than expected. There is a lack of studies linking cancer patients’ vulnerability to rapidly changing security–political situations. In Finland, where military conflicts are decades old, fear of war still impacts mental health, causing depression and anxiety (Hajek et al., 2023). Our study illustrates that dual threats of illness and potential conflict undermine patients’ sense of agency, transforming cancer from a manageable condition to a mentally stressful enemy.

This study offers insights on subjective health experiences and empowerment in the face of severe illness. The retrospective reconstruction of patients’ actions and meanings attributed to cancer can serve as a resource for patients in the future, such as empowering them to share their experiential knowledge as experts with experience (Castro et al., 2019).

Our findings suggest future research should explore various agency-related perspectives, like the sense of control (Pacherie, 2007), and their impact on illness management. Further research should examine how patients with other cancers and serious diseases construct their agency amid contextual, institutional, and social challenges. A notable area is those with extended life expectancies but facing stigmatized understandings (Keum & Giovannucci, 2019; Shim et al., 2021).

## Note

1. During WWII, Finland fought against the Soviet Union in the Winter War in 1939–1940, the Continuation War in 1940–1941, and the Lapland War in 1944–1945. In the end, 100,000 Finns were killed, and Finland lost a tenth of its territory (Nenye et al., 2016).
